# Supplementary material for: Monovalent cations differentially regulate the activity of eukaryotic fluoride channels and impact fluoride resistance
Source: PLoS Biol. 2026 Sep 21;24(9):e3004008. doi: 10.1371/journal.pbio.3004008 (PMC13623104; doi:10.1371/journal.pbio.3004008)
Supplement: Supplementary file 1 [file pbio.3004008.s009.docx]

|  | FEX-CA (EMD-76140)  (PDB 11WR) |
| --- | --- |
| **Data collection and processing** |  |
| Magnification | 105,000 x |
| Voltage (kV)  Bioquantum slit width(eV) | 300  20 |
| Electron exposure (e-/Å^2^) | 50 |
| Defocus range (µm) | -0.8 to -2.5 |
| Pixel size (Å) | 0.834 |
| Symmetry imposed | C1 symmetry |
| Initial particle images (no.) |  |
| Final particle images (no.) | 112,190 |
| Map resolution (Å) | 3.56 |
| Map resolution range (Å) | 3.0 to 8.1 |
| FSC threshold | 0.143 |
| **Refinement** |  |
| Initial model used | PDB 9OP1 |
| Model resolution (Å) | 3.49 |
| FSC threshold | 0.143 |
| Map sharpening B factor (Å^2^) | 137.8 |
| Model-to-map, CC_mask_ | 0.81 |
| Model composition |  |
| Non-hydrogen atoms | 5,843 |
| Protein residues | 766 |
| Water | - |
| B factor (Å^2^) |  |
| Protein | 77.51 |
| Water | - |
| R.m.s. deviations |  |
| Bond lengths (Å) | 0.004 |
| Bond angles (º) | 0.683 |
| **Validation** |  |
| MolProbity score | 2.42 |
| Clashscore | 11.51 |
| Poor rotamers (%) | 3.3 |
| Ramachandran plot |  |
| Favored (%) | 92.5 |
| Allowed (%) | 7.37 |
| Outliers (%) | 0.13 |
