# Supplementary material for: Monovalent cations differentially regulate the activity of eukaryotic fluoride channels and impact fluoride resistance
Source: PLoS Biol. 2026 Sep 21;24(9):e3004008. doi: 10.1371/journal.pbio.3004008 (PMC13623104; doi:10.1371/journal.pbio.3004008)
Supplement: Supplementary file 2 [file pbio.3004008.s013.docx]

**Point-by-point response to comments raised by the reviewers**

Thank you to all three reviewers for their favorable reviews of this work. We have updated the manuscript text to address all of the comments.

**Reviewer #1:**

1. Assignment of the Li+-associated structural state

A central limitation is that Li+ is not directly visualized in the cryo-EM map. The authors explicitly state that, because of the low atomic mass of Li+, they do not expect to observe lithium density within the cation-binding motif (Page 6). I agree that this is technically understandable. However, throughout the manuscript the structure is often referred to as "Li+-FEX-CA" or as a Li+-bound state.

Because the cation itself is not directly observed, the assignment of a Li+-associated state relies on buffer conditions, prior cation-exchange measurements, functional competition experiments, local structural differences, and MD simulations. The authors should make this limitation clearer and use more precise language. For example, "structure under Li+ conditions" would be more accurate unless additional evidence for Li+ occupancy is provided.

**Response**: We have updated the manuscript to clearly acknowledge this limitation. Specifically, we now refer to the structure as “Li⁺-associated FEX-CA,” or “FEX-CA determined in the presence of Li^+^” throughout. We keep Li^+^-FEX-CA and Na^+^-FEX-CA when referring to MD simulation since cations are added to the model for the MD simulations.

We also made a few modifications

Introduction (Page 3, line 83-84):

*The introduction of Li^+^ is associated with a subtle conformational change…*

Result (Page 5, line 159-162):

However, although the conformational differences are modest, we observe displacements of pore-lining helices TM3b and TM4, along with changes to some loop regions for FEX-CA determined under Li^+^ conditions (**Fig 3B**).

2. Ideally, the authors should provide additional support for the cation occupancy under the cryo-EM sample conditions. This could include more quantitative discussion of cation exchange, residual Na+, and expected Li+ occupancy. This is particularly relevant because earlier purification steps include NaCl, whereas the final SEC/grid-preparation condition contains LiCl. If feasible, direct measurement of sample-associated cations by ICP-MS/OES or a related method would substantially strengthen the conclusion. At minimum, the authors should explicitly discuss the possibility of incomplete cation exchange or residual Na+ and explain how this would affect the interpretation of the observed F159 rearrangement and pore constriction.

**Response**: Unfortunately, we no longer have the protein sample that we used for cryo-EM to analyze using ICP-MS. We have added an estimate of residual Na^+^ and cation occupancy for our cryo-EM sample in the *Methods* section. Given the large excess of Li^+^ and the time provided for cation exchange (5 hours) we expect Li^+^ to be the major bound cation. We have modified the text in two places to describe this:

Page 5, line 178-181:

*To prepare the Li^+^-associated FEX-CA sample, 150 mM LiCl was added in lieu of NaCl during the final step of protein purification and incubated with the sample for 5 hours prior to freezing the grids. A prior measurement of the cation exchange kinetics (t_1/2_=2h) indicate that Li^+^ should be the major bound cation with ~92% occupancy under these conditions.*

Material and Methods section (Page 14, line 623-627):

We added the following text to the section *Cryo-EM grid preparation and data collection*

*We previously showed that Li^+^/Na^+^ exchange is a first order process with a t_1/2_ of 2 hours in membranes. After buffer exchange into the 150 mM Li^+^ solution, we allowed 5 hours before freezing grids. Given these parameters, we expect Li^+^ occupancy to be 92% and remaining Na^+^ occupancy to be 8%. On the background of 150 mM Li^+^, contamination by up to 5 mM Na^+^ is not expected to significantly compete with Li^+^ for occupancy of the binding site.*

3. Yeast growth experiment

The yeast growth experiment shows that LiCl reduces growth in the presence of fluoride. This is consistent with FEX inhibition, but it does not establish that the effect is mediated through FEX. The current assay uses wild-type S. cerevisiae, and the authors report that KF alone does not affect growth, whereas LiCl plus KF reduces growth in a dose-dependent manner. The Methods also state that the assay used wild-type S. cerevisiae BY4741, and the Figure 2 legend describes wild-type cells.

This experiment does not distinguish direct FEX inhibition from broader cellular effects of Li+. This is particularly important because the authors' previous work used a deltafex1deltafex2 complementation system to evaluate FEX function in yeast. A similar genetic approach would be highly appropriate here. If such experiments are not added, the relevant statements should be softened.

**Response:** We do agree that using *∆fex1∆fex2* and our galactose-induced complementation system might, in principle, be a better system for these experiments. In fact, we tried this first. However, it turns out that Li^+^ is specifically toxic to *S. cerevisiae* in galactose-containing media (Masuda, Previato *et al.* 2008), since it causes the accumulation of toxic intermediates in the galactose metabolism pathway. This precludes use of the *∆fex1∆fex2* complementation system, which requires galactose to induce protein expression. So, this approach, while a good idea, does not work for this technical reason.

We nonetheless think that it is unlikely that the inhibition we see is due to broader cellular effects of Li^+^. As we show in Figure 2, Li^+^ alone causes no detectable growth defect up to 100 mM in the absence of fluoride, whereas we already see inhibition with 3 mM Li^+^ when fluoride is present. Since FEX is basically the sole determinant of fluoride resistance in yeast, it seems very unlikely that some other cellular target is responsible for this phenotype, which only emerges when both Li^+^ and F^-^ are present. However, since we can’t formally exclude the possibility, we have changed the text as follows:

Results (Page 4, line 142-149):

*“These experiments show that Li^+^ diminishes the ability of eukaryotes to withstand fluoride, as predicted by the in vitro observation of FEX inhibition by Li^+^…. These experiments do not formally exclude indirect effects of Li^+^ on fluoride sensitivity, but FEX is by far the dominant mechanism of fluoride resistance in yeast, and Li^+^ is otherwise non-toxic to yeast in rich media, even at high concentrations[19].*

4. Physiological and applied significance of Li+ inhibition

The physiological significance of Na+-dependent activation is relatively clear, because Na+ is biologically abundant and the reported affinity is in a plausible physiological range. In contrast, the physiological relevance of Li+-dependent inhibition is less well established. The yeast phenotype is observed at relatively high LiCl concentrations, and the manuscript does not show that Li+ reaches inhibitory concentrations near FEX under natural cellular or environmental conditions.

Thus, Li+ inhibition should be described more cautiously as a potential environmental or toxicological interaction with fluoride stress, rather than as a broadly physiological regulatory mechanism. Similarly, the study may provide a useful mechanistic hint for inhibitor development by identifying an inactive, F159-constricted state, but Li+ itself is unlikely to be a selective or practical FEX inhibitor. Any statements implying antifungal or inhibitor-development relevance should be presented as a possible implication, not as an established application.

**Response:** We did not intend to imply that Li^+^ is physiological regulator of FEX, except so far as that Li^+^ is found in some environments, just as fluoride is, and that the effective resistance to either ion will be impacted by the presence of the other. Thinking about the microbial resistance literature more broadly, I think this is an important point to make, since these resistances are rarely studied in combination, although the interplay between them might be complex.

To ensure that we are clear about this point, we have changed instances in the abstract and discussion where we describe Li^+^ as “relevant *in vivo*” to “environmentally relevant” and given a couple of examples of natural environments with Li^+^ concentrations in the tens of millimolar range (for example marine sediments have up to 40 mM Li+, line 536 in the discussion).

We added the following to expand on this idea (line 559-561):

*These results provide another example of the complex interplay between adaptations to different salts[38]. For organisms in environments where lithium and fluoride co-occur, our results provide a mechanistic foundation to understand the relationship between environmental chemistry and fluoride stress tolerance.*

The reviewer is also correct that Li^+^ is not a very potent inhibitor. We don’t ever refer to it as an “antifungal” in this text, and when we call it an inhibitor or an antagonist, we only use the word in the biochemical sense, since its addition undoubtedly inhibits channel activity.

5. In the Abstract, the phrase "and the alter the rotamer" should be corrected to "and alter the rotamer" or a similar grammatical construction (Page 1, Lines 39-40).

**Response:** Thank you!. We’ve corrected it in the revised manuscript. (Page 1, line 39)

6. The Figure 2 legend contains "grown in in the absence", which should be corrected (Page 24, Lines 765-768).

**Response:** Thank you! We’ve corrected it in the revised manuscript.

**Reviewer #2:**

The most critical weakness of the study is the ability to distinguish subtle structural differences between FEX in Na+ and Li+. On page 5 the authors acknowledge that the differences are subtle (line 149) but then go on to explain the differences with apparent confidence. To me the structural differences in the two sets of maps are not as solid as the text would have the reader to believe and I would like to see the authors present the differences more cautiously. All of the differences in Fig. 4A and B are quite small given the resolution of the two structures and I would like the text to reflect this uncertainty. Very small repositioning of side chains could easily yield the differences in the HOLE plot shown in Fig 4B. Also, the presentation of maps in Supplementary Fig 4 should be moved to Fig.4 and perhaps improved to help the reader see the subtle changes observed. (I believe the authors mixed up the Left and Right in the legend to Fig. S4). Unless I missed it, it would be good to remind the reader that the binding sites for both Na+ and Li+ have yet to be resolved in FEX, and it might be good to repeat the rationale from their earlier paper (ref 9) where they use a structure of FLUC with Na+ bound to make a case for where the cations might bind in FEX.

**Response:** While we agree that the structures should be interpreted cautiously, we note that our structural interpretations are bolstered by an independent method, as the MD simulations, initiated from the original Na^+^ structure, converged on similar changes to the S159 and F159 region in the presence of Li^+^. We have updated the text throughout to better convey the structural uncertainties, and, as requested, we have moved the maps to main Fig 4. Thanks also for catching the mistake in the Figure S4 legend. That is now corrected. Changes include the following:

Results (Page 5-6):

*However, although the conformational differences are modest, we observe displacements of pore-lining helices TM3b and TM4, along with changes to some loop regions for FEX-CA determined under Li^+^ conditions (****Fig 3B****).*

*Although these differences are subtle at this resolution, the reduction in pore radius consistent with our functional observation of FEX inhibition by Li^+^.*

*However, this site exhibits a high degree of structural similarity throughout the Fluc/FEX family, and computational studies demonstrated stable sodium binding at this site in FEX-CA[9, 23]. Although the cation itself is not resolved…*

*We propose that the vestibule-facing interaction between S158 and S161 perturbs the local backbone geometry of the TM3 break (which is not constrained by α-helical secondary structure) including pore-lining F159*

**Reviewer #3:**

1. To me, the role of the positively charged R89 in the binding of the cations Na+ and Li+ is not entirely clear. Is charge repulsion involved in the subtle structural movement of FEX?

**Response:** In both our cryo-EM structure and MD simulation results, R89 remains in a similar position. We use it as a point of reference, since it stays at a consistent position, and S158 rotamerizes to be within hydrogen bonding distance of R89 in the Na^+^-associated FEX, (Fig. 5C, Fig. S5). Since we don’t observe a significant change in the sidechain of R89 between Na^+^ and Li^+^ conditions, we think that the local arrangement of the adjacent F159 is likely driven by a rotamer change of S158 rather than electrostatically influenced by R89. We agree that the cation binding site is quite close to the R89, but this is seen in the Flucs as well, and there are also plenty of anionic sidechains in the region.

To clarify our thoughts about R89 in the text, we have updated the text as follows:

Result (Page 6, line 341-344):
*S158 rotates away from its hydrogen bonding partner, R89, in the Na^+^-associated FEX-CA structure, and instead moves to within hydrogen bonding distance of the S161 sidechain in the Li^+^-associated FEX-CA structure (****S4A, S5 Figs)****. R89 remains in the same position relative to the crossover region in the two structures.”*

2. The flux assays in Fig. 1A presents data for 30 mM Na+ at 0.6 and 2 mM Li+, which appear to be omitted in Fig. 1C. Is there any convincing justification? If so, please comment. Furthermore, the data points will increase the precision of EC50 determination by the dose-response data in Fig. 1C.

**Response:** The data points for 0.6 and 2 mM Li^+^ at 30 mM Na^+^ were present but hidden by overlapping datapoints. We’ve revised Fig 1C to use smaller datapoints with distinct shapes (triangles, circles, and squares).

3. The state transitions depicted in Fig. 1B could be incorporated into one of the subfigures.

**Response:** Thank you for the suggestion. We decided to keep this scheme in Fig 1B to fill the available space and provide a clear interpretation of our model for cation exchange.

4. In Fig. 2, the amount of Li+ is several orders of magnitude higher than the Kd that was obtained by the flux assays. Following this, I would expect complete loss of fluoride resistance at already 3 and 10 mM Li+.

**Response:** Yes, in our reconstituted system, we extrapolate the Li^+^ K_d_ to be ~200 μM in the absence of any other cations. In our growth experiments, half-maximal inhibition occurs in the 5-10 mM range. We have two thoughts on why this might be: 1) Whereas we can prepare reconstituted systems with well-defined concentration of each monovalent cation, we don’t believe that these concentrations are as well-controlled in the growth experiments. As competitive agonists, biologically abundant cations K^+^ and especially Na^+^ will reduce the apparent K_d_ for Li^+^ in the growth experiments. The presence of just 3 mM Na^+^ near the plasma membrane would explain most of the discrepancy. 2) Reconstituted transport experiments used *Candida albicans* FEX, whereas the yeast growth assay was performed using *Saccharomyces cerevisiae*, so the binding constants might differ slightly between the two species.

We have added the following text (line 144-147):

*Although the apparent inhibitory concentration of Li^+^ is higher than the K_d_ determined in the reconstituted system for the plant and C. albicans homologues, interference by biological cations K^+^ (included at 50 mM in the media) and Na^+^ is expected to reduce the effective inhibitory concentration.*

5. I recommend including the boundaries of the lipid bilayer to Fig. 4A and B, similar as presented in Fig. 3A.

**Response:** Thank you for the suggestion. We’ve added lipid boundaries for Fig 4B.
