# Supplementary material for: Monovalent cations differentially regulate the activity of eukaryotic fluoride channels and impact fluoride resistance
Source: PLoS Biol. 2026 Sep 21;24(9):e3004008. doi: 10.1371/journal.pbio.3004008 (PMC13623104; doi:10.1371/journal.pbio.3004008)
Supplement: Peer review history

# Editor report: Decision Letter 0

**DOI:** 10.1371/journal.pbio.3004008.r001

**Authors:** Richard Hodge

3 Jun 2026

Dear Dr Stockbridge,

Thank you for submitting your manuscript entitled "The structural mechanism of eukaryotic fluoride channel activation and inhibition by monovalent cations" for consideration as a Research Article by PLOS Biology.

Your manuscript has now been evaluated by the PLOS Biology editorial staff and I am writing to let you know that we would like to send your submission out for external peer review.

However, before we can send your manuscript to reviewers, we need you to complete your submission by providing the metadata that is required for full assessment. To this end, please login to Editorial Manager where you will find the paper in the 'Submissions Needing Revisions' folder on your homepage. Please click 'Revise Submission' from the Action Links and complete all additional questions in the submission questionnaire.

Once your full submission is complete, your paper will undergo a series of checks in preparation for peer review. After your manuscript has passed the checks it will be sent out for review. To provide the metadata for your submission, please Login to Editorial Manager (https://www.editorialmanager.com/pbiology) within two working days, i.e. by Jun 05 2026 11:59PM.

If your manuscript has been previously peer-reviewed at another journal, PLOS Biology is willing to work with those reviews in order to avoid re-starting the process. Submission of the previous reviews is entirely optional and our ability to use them effectively will depend on the willingness of the previous journal to confirm the content of the reports and share the reviewer identities. Please note that we reserve the right to invite additional reviewers if we consider that additional/independent reviewers are needed, although we aim to avoid this as far as possible. In our experience, working with previous reviews does save time.

If you would like us to consider previous reviewer reports, please edit your cover letter to let us know and include the name of the journal where the work was previously considered and the manuscript ID it was given. In addition, please upload a response to the reviews as a 'Prior Peer Review' file type, which should include the reports in full and a point-by-point reply detailing how you have or plan to address the reviewers' concerns.

During the process of completing your manuscript submission, you will be invited to opt-in to posting your pre-review manuscript as a bioRxiv preprint. Visit http://journals.plos.org/plosbiology/s/preprints for full details. If you consent to posting your current manuscript as a preprint, please upload a single Preprint PDF.

Feel free to email us at plosbiology@plos.org if you have any queries relating to your submission.

Kind regards,

Richard

Richard Hodge, PhD

Senior Editor, PLOS Biology

rhodge@plos.org

PLOS

Empowering researchers to transform science

Carlyle House, Carlyle Road, Cambridge, CB4 3DN, United Kingdom

California (U.S.) corporation #C2354500, based in San Francisco

# Peer review: Decision Letter 1

**DOI:** 10.1371/journal.pbio.3004008.r002

**Authors:** Richard Hodge

23 Jul 2026

Dear Dr Stockbridge,

Thank you for your patience while your manuscript "The structural mechanism of eukaryotic fluoride channel activation and inhibition by monovalent cations" was peer-reviewed at PLOS Biology. Please accept my sincere apologies for the delays that you have experienced during the peer review process. Your manuscript has now been evaluated by the PLOS Biology editors, an Academic Editor with relevant expertise, and by three independent reviewers.

In light of the reviews, which you will find at the end of this email, we would like to invite you to revise the work to thoroughly address the reviewers' reports.

As you will see below, the reviewers are generally positive about your manuscript and think it is interesting and well done. Reviewer #1 raises concerns about the assignment of the Li+-associated structural state, yeast growth experiments and the physiological relevance of Li+-dependent inhibition. The reviewer asks that additional experiments are included to provide additional support for the findings. In addition, Reviewer #2 raises concerns about the ability to distinguish subtle structural differences between FEX in Na+ and Li+ and requests that the conclusions are tempered. Finally, Reviewer #3 provides a few comments to improve the data presentation and reporting.

Given the extent of revision needed, we cannot make a decision about publication until we have seen the revised manuscript and your response to the reviewers' comments. Your revised manuscript is likely to be sent for further evaluation by all or a subset of the reviewers.

In addition to these revisions, you will need to complete some formatting changes, which you will receive in a follow up email. A member of our team will be in touch with a set of requests shortly.

We expect to receive your revised manuscript within 3 months. Please email us (plosbiology@plos.org) if you have any questions or concerns, or would like to request an extension.

At this stage, your manuscript remains formally under active consideration at our journal; please notify us by email if you do not intend to submit a revision so that we may withdraw it.

**IMPORTANT - SUBMITTING YOUR REVISION**

Your revisions should address the specific points made by each reviewer. Please submit the following files along with your revised manuscript:

1. A 'Response to Reviewers' file - this should detail your responses to the editorial requests, present a point-by-point response to all of the reviewers' comments, and indicate the changes made to the manuscript.

*NOTE: In your point-by-point response to the reviewers, please provide the full context of each review. Do not selectively quote paragraphs or sentences to reply to. The entire set of reviewer comments should be present in full and each specific point should be responded to individually, point by point.

You should also cite any additional relevant literature that has been published since the original submission and mention any additional citations in your response.

2. In addition to a clean copy of the manuscript, please also upload a 'track-changes' version of your manuscript that specifies the edits made. This should be uploaded as a "Revised Article with Changes Highlighted" file type.

*Re-submission Checklist*

When you are ready to resubmit your revised manuscript, please refer to this re-submission checklist: https://plos.io/Biology_Checklist

To submit a revised version of your manuscript, please go to https://www.editorialmanager.com/pbiology/ and log in as an Author. Click the link labelled 'Submissions Needing Revision' where you will find your submission record.

Please make sure to read the following important policies and guidelines while preparing your revision:

*Published Peer Review*

Please note while forming your response, if your article is accepted, you may have the opportunity to make the peer review history publicly available. The record will include editor decision letters (with reviews) and your responses to reviewer comments. If eligible, we will contact you to opt in or out. Please see here for more details:

https://blogs.plos.org/plos/2019/05/plos-journals-now-open-for-published-peer-review/

*PLOS Data Policy*

Please note that as a condition of publication PLOS' data policy (http://journals.plos.org/plosbiology/s/data-availability) requires that you make available all data used to draw the conclusions arrived at in your manuscript. If you have not already done so, you must include any data used in your manuscript either in appropriate repositories, within the body of the manuscript, or as supporting information (N.B. this includes any numerical values that were used to generate graphs, histograms etc.). For an example see here: http://www.plosbiology.org/article/info%3Adoi%2F10.1371%2Fjournal.pbio.1001908#s5

*Blot and Gel Data Policy*

We require the original, uncropped and minimally adjusted images supporting all blot and gel results reported in an article's figures or Supporting Information files. We will require these files before a manuscript can be accepted so please prepare them now, if you have not already uploaded them. Please carefully read our guidelines for how to prepare and upload this data: https://journals.plos.org/plosbiology/s/figures#loc-blot-and-gel-reporting-requirements

*Protocols deposition*

To enhance the reproducibility of your results, we recommend that if applicable you deposit your laboratory protocols in protocols.io, where a protocol can be assigned its own identifier (DOI) such that it can be cited independently in the future. Additionally, PLOS ONE offers an option for publishing peer-reviewed Lab Protocol articles, which describe protocols hosted on protocols.io. Read more information on sharing protocols at https://plos.org/protocols?utm_medium=editorial-email&utm_source=authorletters&utm_campaign=protocols

Thank you again for your submission to our journal. We hope that our editorial process has been constructive thus far, and we welcome your feedback at any time. Please don't hesitate to contact us if you have any questions or comments.

Best wishes,

Richard

Richard Hodge, PhD

Senior Editor, PLOS Biology

rhodge@plos.org

------------------------------------

REVIEWS:

Reviewer #1: In this manuscript, the authors investigate how monovalent cations regulate the eukaryotic fluoride channel FEX. The authors show that Li+ inhibits Na+-dependent FEX activity in reconstituted liposomes and propose that Li+ acts as a competitive antagonist at the central cation-binding site. They further report that LiCl reduces fluoride tolerance in yeast, determine a cryo-EM structure of C. albicans FEX under LiCl conditions, and use MD simulations to compare Na+- and Li+-associated states. The central model is that Li+ favors a more compact cation-binding geometry and promotes structural rearrangements that stabilize a narrowed pore.

Notably, the manuscript builds substantially on the authors' recent work on Na+-dependent FEX structure and function (Nat Commun 2026, PMID: 41381451). This is evident in the Introduction and Results. The new contribution of the present manuscript is the mechanistic extension to Li+ inhibition. This is potentially interesting and would be a valuable follow-up to the prior work.

Overall, this is a technically solid study. The combination of reconstituted transport assays, cryo-EM, yeast growth assays, and MD simulations is appropriate. However, several aspects of the current manuscript require clarification or additional support before the main conclusions are fully justified.

Major comments

1. Assignment of the Li+-associated structural state

A central limitation is that Li+ is not directly visualized in the cryo-EM map. The authors explicitly state that, because of the low atomic mass of Li+, they do not expect to observe lithium density within the cation-binding motif (Page 6). I agree that this is technically understandable. However, throughout the manuscript the structure is often referred to as "Li+-FEX-CA" or as a Li+-bound state.

Because the cation itself is not directly observed, the assignment of a Li+-associated state relies on buffer conditions, prior cation-exchange measurements, functional competition experiments, local structural differences, and MD simulations. The authors should make this limitation clearer and use more precise language. For example, "structure under Li+ conditions" would be more accurate unless additional evidence for Li+ occupancy is provided.

Ideally, the authors should provide additional support for the cation occupancy under the cryo-EM sample conditions. This could include more quantitative discussion of cation exchange, residual Na+, and expected Li+ occupancy. This is particularly relevant because earlier purification steps include NaCl, whereas the final SEC/grid-preparation condition contains LiCl. If feasible, direct measurement of sample-associated cations by ICP-MS/OES or a related method would substantially strengthen the conclusion. At minimum, the authors should explicitly discuss the possibility of incomplete cation exchange or residual Na+ and explain how this would affect the interpretation of the observed F159 rearrangement and pore constriction.

2. Yeast growth experiment

The yeast growth experiment shows that LiCl reduces growth in the presence of fluoride. This is consistent with FEX inhibition, but it does not establish that the effect is mediated through FEX. The current assay uses wild-type S. cerevisiae, and the authors report that KF alone does not affect growth, whereas LiCl plus KF reduces growth in a dose-dependent manner. The Methods also state that the assay used wild-type S. cerevisiae BY4741, and the Figure 2 legend describes wild-type cells.

This experiment does not distinguish direct FEX inhibition from broader cellular effects of Li+. This is particularly important because the authors' previous work used a deltafex1deltafex2 complementation system to evaluate FEX function in yeast. A similar genetic approach would be highly appropriate here. If such experiments are not added, the relevant statements should be softened.

3. Physiological and applied significance of Li+ inhibition

The physiological significance of Na+-dependent activation is relatively clear, because Na+ is biologically abundant and the reported affinity is in a plausible physiological range. In contrast, the physiological relevance of Li+-dependent inhibition is less well established. The yeast phenotype is observed at relatively high LiCl concentrations, and the manuscript does not show that Li+ reaches inhibitory concentrations near FEX under natural cellular or environmental conditions.

Thus, Li+ inhibition should be described more cautiously as a potential environmental or toxicological interaction with fluoride stress, rather than as a broadly physiological regulatory mechanism. Similarly, the study may provide a useful mechanistic hint for inhibitor development by identifying an inactive, F159-constricted state, but Li+ itself is unlikely to be a selective or practical FEX inhibitor. Any statements implying antifungal or inhibitor-development relevance should be presented as a possible implication, not as an established application.

Minor comments

1. In the Abstract, the phrase "and the alter the rotamer" should be corrected to "and alter the rotamer" or a similar grammatical construction (Page 1, Lines 39-40).

2. The Figure 2 legend contains "grown in in the absence", which should be corrected (Page 24, Lines 765-768).

Reviewer #2: This is an interesting manuscript describing the mechanism of inhibition of the eukaryotic FEX fluoride exporter by Li+ ions. A recent structure of FEX has revealed their structural relationship with the more extensively studied prokaryotic FLUC fluoride exporters, both of which require a Na+ ion bound for fluoride efflux. Previous structures of FLUC have revealed where Na+ ions bind so tightly that they can only be exchanges on the timescale of days, whereas previous structures of FEX failed to resolve where Na+ ions bind, although computational efforts support the binding at a site in FEX similar to that seen in FLUCs. FLUCs are homodimers arranged in an antiparallel fashion, whereas FEX are monomeric containing two homologous antiparallel domains that form one fluoride efflux pathway and one vestigial pathway. In the present study, the authors use a fluoride efflux assay and Schild analysis to show that Li+ inhibits FEX through a mechanism that is competitive with Na+ (Fig.1), with an affinity of 180 nM. The authors then show that Li+ inhibits the grown the yeast in the presence of fluoride (where FEX activity is required for survival) but not in the absence of fluoride, implicating FEX in the inhibitory mechanism for Li+. Next, the authors solve a cryo-EM structure of FEX at 3.5 Å in the presence of 150 mM Li+ and observe subtle structural changes near the middle of the membrane spanning fluoride permeation pathway in the vicinity of F159 and I163 that appear to narrow the fluoride permeation pathway. Finally, the authors undertake quite substantial 0.75 µs MD simulations on the FEX structure in Li+ and compare their results with their previous simulations of their earlier structure solved in Na+. These simulations support the subtle structural differences between FEX with Na+ and Li+ bound and provide a plausible mechanism for Li+ inhibition of the fluoride exporter. I like the study and my only reservation is the confidence with which the structural differences between FEX with Na+ and Li+ bound are presented (or at least perceived by this reviewer).

The most critical weakness of the study is the ability to distinguish subtle structural differences between FEX in Na+ and Li+. On page 5 the authors acknowledge that the differences are subtle (line 149) but then go on to explain the differences with apparent confidence. To me the structural differences in the two sets of maps are not as solid as the text would have the reader to believe and I would like to see the authors present the differences more cautiously. All of the differences in Fig. 4A and B are quite small given the resolution of the two structures and I would like the text to reflect this uncertainty. Very small repositioning of side chains could easily yield the differences in the HOLE plot shown in Fig 4B. Also, the presentation of maps in Supplementary Fig 4 should be moved to Fig.4 and perhaps improved to help the reader see the subtle changes observed. (I believe the authors mixed up the Left and Right in the legend to Fig. S4). Unless I missed it, it would be good to remind the reader that the binding sites for both Na+ and Li+ have yet to be resolved in FEX, and it might be good to repeat the rationale from their earlier paper (ref 9) where they use a structure of FLUC with Na+ bound to make a case for where the cations might bind in FEX.

Reviewer #3: The manuscript from Kang et al. analyses the ligand-gated, fluoride channel FEX by Na+ and Li+, which activate and inhibit the fluoride transport, respectively. Since fluoride accumulation in cells is toxic, FEX activity is crucial for fluoride resistance in eukaryotes. The authors have previously published an article that focuses on the mechanism of FEX activation by Na+ (doi: 10.1038/s41467-025-67289-0). The current manuscript is a follow up study, in which the authors focus on the mechanism of the Li+-mediated inhibition. The implementation of complementary approaches renders the study appealing and follows the combination of methods used in their previous manuscript. Overall, the study presents a significant advancement in understanding the mechanism and regulation of FEX and fluoride resistance in eukaryotes. However, data presentation could be improved to enhance the clarity of the results.

I want to bring up the following comments:

1.To me, the role of the positively charged R89 in the binding of the cations Na+ and Li+ is not entirely clear. Is charge repulsion involved in the subtle structural movement of FEX?

2.The flux assays in Fig. 1A presents data for 30 mM Na+ at 0.6 and 2 mM Li+, which appear to be omitted in Fig. 1C. Is there any convincing justification? If so, please comment. Furthermore, the data points will increase the precision of EC50 determination by the dose-response data in Fig. 1C.

3.The state transitions depicted in Fig. 1B could be incorporated into one of the subfigures.

4.In Fig. 2, the amount of Li+ is several orders of magnitude higher than the Kd that was obtained by the flux assays. Following this, I would expect complete loss of fluoride resistance at already 3 and 10 mM Li+.

5.I recommend including the boundaries of the lipid bilayer to Fig. 4A and B, similar as presented in Fig. 3A.

# Author comment: Author response to Decision Letter 2

**DOI:** 10.1371/journal.pbio.3004008.r003

20 Aug 2026

Attachment Submitted filename: response_to_reviewers_PlosBiology.docx

# Editor report: Decision Letter 2

**DOI:** 10.1371/journal.pbio.3004008.r004

**Authors:** Richard Hodge

1 Sep 2026

Dear Dr Stockbridge,

Thank you for your patience while we considered your revised manuscript "The structural mechanism of eukaryotic fluoride channel activation and inhibition by monovalent cations" for publication as a Research Article at PLOS Biology. This revised version of your manuscript has been evaluated by the PLOS Biology editors and the Academic Editor.

Based on our Academic Editor's assessment of your revision, I am pleased to say that we are likely to accept this manuscript for publication, provided you satisfactorily address the following data and other policy-related requests that I have provided below (A-F):

(A) We routinely suggest changes to titles to ensure maximum accessibility for a broad, non-specialist readership. In this case, we would like to suggest the following few titles, which are a bit more declarative and provide additional information on the main findings of the study. We would also welcome any suggestions that you may have along these lines. Please ensure you change both the manuscript file and the online submission system, as they need to match for final acceptance:

“Monovalent cations differentially regulate the activity of eukaryotic fluoride exporters and impact fluoride resistance"

“Lithium antagonizes sodium-dependent activation of eukaryotic fluoride exporters”

(B) Thank you for already providing the underlying data for Figures 1A, 1C-D and 2A-C in the source data file. After reviewing Figure 5, I wonder whether some underlying data is missing in the file for this figure, especially Figure 5B and 5E? If so, I would be grateful if you could please update the source data file and include.

(C) Please also ensure that each of the relevant figure legends in your manuscript include information on *WHERE THE UNDERLYING DATA CAN BE FOUND*, and ensure your supplemental data file/s has a legend.

(D) Please ensure that your Data Statement in the submission system accurately describes where your data can be found and is in final format, as it will be published as written there.

(E) Please add the links to the funding agencies in the Financial Disclosure statement in the manuscript details.

(F) Per journal policy, if you have generated any custom code during the course of this investigation, please make it available without restrictions. Please ensure that the code is sufficiently well documented and reusable, and that your Data Statement in the Editorial Manager submission system accurately describes where your code can be found. More information on our Code Policy, what and how to share can be found here: https://journals.plos.org/plosbiology/s/code-availability

Please note that we cannot accept sole deposition of code in GitHub, as this could be changed after publication. However, you can archive this version of your publicly available GitHub code to Zenodo. Once you do this, it will generate a DOI number, which you will need to provide in the Data Accessibility Statement (you are welcome to also provide the GitHub access information). See the process for doing this here: https://docs.github.com/en/repositories/archiving-a-github-repository/referencing-and-citing-content

------------------------------------------------------------------------

As you address these items, please take this last chance to review your reference list to ensure that it is complete and correct. If you have cited papers that have been retracted, please include the rationale for doing so in the manuscript text, or remove these references and replace them with relevant current references. Any changes to the reference list should be mentioned in the cover letter that accompanies your revised manuscript.

In addition to these revisions, you may need to complete some formatting changes, which you will receive in a follow up email. A member of our team will be in touch with a set of requests shortly. If you do not receive a separate email within a few days, please assume that checks have been completed, and no additional changes are required.

We expect to receive your revised manuscript within two weeks.

To submit your revision, please go to https://www.editorialmanager.com/pbiology/ and log in as an Author. Click the link labelled 'Submissions Needing Revision' to find your submission record. Your revised submission must include the following:

- a cover letter that should detail your responses to any editorial requests, if applicable, and whether changes have been made to the reference list

- a Response to Reviewers file that provides a detailed response to the reviewers' comments (if applicable, if not applicable please do not delete your existing 'Response to Reviewers' file.)

- a track-changes file indicating any changes that you have made to the manuscript.

NOTE: If Supporting Information files are included with your article, note that these are not copyedited and will be published as they are submitted. Please ensure that these files are legible and of high quality (at least 300 dpi) in an easily accessible file format. For this reason, please be aware that any references listed in an SI file will not be indexed. For more information, see our Supporting Information guidelines:

https://journals.plos.org/plosbiology/s/supporting-information

*Published Peer Review History*

Please note that you may have the opportunity to make the peer review history publicly available. The record will include editor decision letters (with reviews) and your responses to reviewer comments. If eligible, we will contact you to opt in or out. Please see here for more details:

https://plos.org/published-peer-review-history/

*Press*

Should you, your institution's press office or the journal office choose to press release your paper, please ensure you have opted out of Early Article Posting on the submission form. We ask that you notify us as soon as possible if you or your institution is planning to press release the article.

*Protocols deposition*

To enhance the reproducibility of your results, we recommend that if applicable you deposit your laboratory protocols in protocols.io, where a protocol can be assigned its own identifier (DOI) such that it can be cited independently in the future. Additionally, PLOS ONE offers an option for publishing peer-reviewed Lab Protocol articles, which describe protocols hosted on protocols.io. Read more information on sharing protocols at https://plos.org/protocols?utm_medium=editorial-email&utm_source=authorletters&utm_campaign=protocols

Please do not hesitate to contact me should you have any questions.

Best regards,

Richard

Richard Hodge, PhD

Senior Editor, PLOS Biology

rhodge@plos.org

PLOS

Empowering researchers to transform science

Carlyle House, Carlyle Road, Cambridge, CB4 3DN, United Kingdom

California (U.S.) corporation #C2354500, based in San Francisco

# Author comment: Author response to Decision Letter 3

**DOI:** 10.1371/journal.pbio.3004008.r005

4 Sep 2026

Attachment Submitted filename: response_to_reviewers_PlosBiology_auresp_3.docx

# Editor report: Decision Letter 3

**DOI:** 10.1371/journal.pbio.3004008.r006

**Authors:** Richard Hodge

7 Sep 2026

Dear Dr Stockbridge,

On behalf of my colleagues and the Academic Editor, Raimund Dutzler, I am pleased to say that we can accept your manuscript for publication, provided you address any remaining formatting and reporting issues. These will be detailed in an email you should receive within 2-3 business days from our colleagues in the journal operations team; no action is required from you until then. Please note that we will not be able to formally accept your manuscript and schedule it for publication until you have completed any requested changes.

Please take a minute to log into Editorial Manager at http://www.editorialmanager.com/pbiology/, click the "Update My Information" link at the top of the page, and update your user information to ensure an efficient production process.

PRESS

We frequently collaborate with press offices. If your institution or institutions have a press office, please notify them about your upcoming paper at this point, to enable them to help maximise its impact. If the press office is planning to promote your findings, we would be grateful if they could coordinate with biologypress@plos.org. If you have previously opted in to the early version process, we ask that you notify us immediately of any press plans so that we may opt out on your behalf.

We also ask that you take this opportunity to read our Embargo Policy regarding the discussion, promotion and media coverage of work that is yet to be published by PLOS. As your manuscript is not yet published, it is bound by the conditions of our Embargo Policy. Please be aware that this policy is in place both to ensure that any press coverage of your article is fully substantiated and to provide a direct link between such coverage and the published work. For full details of our Embargo Policy, please visit http://www.plos.org/about/media-inquiries/embargo-policy/.

Thank you again for choosing PLOS Biology for publication and supporting Open Access publishing. We look forward to publishing your study.

Best wishes,

Richard

Richard Hodge, PhD

Senior Editor, PLOS Biology

rhodge@plos.org

PLOS

Empowering researchers to transform science

Carlyle House, Carlyle Road, Cambridge, CB4 3DN, United Kingdom

California (U.S.) corporation #C2354500, based in San Francisco
